# Therapeutic potential of resveratrol through ferroptosis modulation: insights and future directions in disease therapeutics

**DOI:** 10.3389/fphar.2024.1473939

**Published:** 2024-09-25

**Authors:** Liu Peng, Xi-Zhuo Hu, Zhi-Qiang Liu, Wen-Kai Liu, Qun Huang, Yue Wen

**Affiliations:** ^1^ Division of Gastrointestinal Surgery, Department of General Surgery, West China Hospital, Sichuan University, Chengdu, China; ^2^ School of Pharmacy, Chengdu University of Traditional Chinese Medicine, Chengdu, China; ^3^ Department of General Surgery, Deyang Sixth People’s Hospital, Deyang, China; ^4^ Department of Ophthalmology, Hospital of Chengdu University of Traditional Chinese Medicine, Chengdu, China

**Keywords:** resveratrol, ferroptosis, lipid peroxidation, cerebrovascular diseases, cardiovascular diseases, cancer

## Abstract

Resveratrol, a naturally occurring polyphenolic compound, has captivated the scientific community with its promising therapeutic potential across a spectrum of diseases. This review explores the complex role of resveratrol in modulating ferroptosis, a newly identified form of programmed cell death, and its potential implications for managing cardiovascular and cerebrovascular disorders, cancer, and other conditions. Ferroptosis is intricately linked to the pathogenesis of diverse diseases, with resveratrol exerting multifaceted effects on this process. It mitigates ferroptosis by modulating lipid peroxidation, iron accumulation, and engaging with specific cellular receptors, thereby manifesting profound therapeutic benefits in cardiovascular and cerebrovascular conditions, as well as oncological settings. Moreover, resveratrol’s capacity to either suppress or induce ferroptosis through the modulation of signaling pathways, including Sirt1 and Nrf2, unveils novel therapeutic avenues. Despite resveratrol’s limited bioavailability, advancements in molecular modification and drug delivery optimization have amplified its clinical utility. Future investigations are poised to unravel the comprehensive mechanisms underpinning resveratrol’s action and expand its therapeutic repertoire. We hope this review could furnish a detailed and novel insight into the exploration of resveratrol in the regulation of ferroptosis and its therapeutic prospects.

## Graphical Abstract

**Figure:**
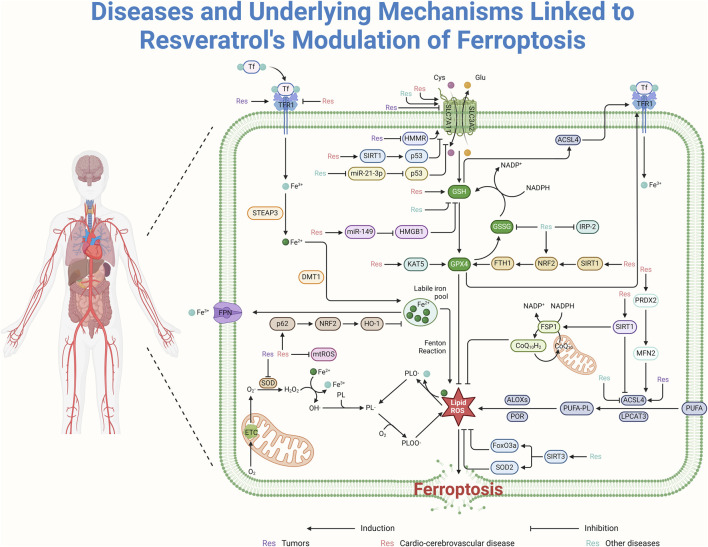
Exploring resveratrol - a novel insight into ferroptosis regulation and therapeutic prospects.

## 1 Introduction

**FIGURE 1:**
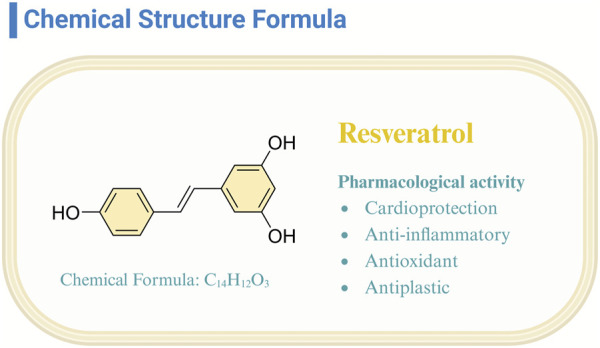
Structure and pharmacological function of resveratrol.

Resveratrol (Res), classified as a non-flavonoid polyphenolic compound, emerges as a phytoalexin produced by plants, notably present in red wine, grapes, and raspberries (Koushki et al., 2018). Its trans isomer variant, distinguished by heightened biological activity and stability, has garnered substantial scientific interest (Pangeni et al., 2014; Riccio et al., 2020). Investigations have elucidated resveratrol’s multifaceted bioactive properties (Mohammadipoor et al., 2022; Vajdi et al., 2023; Wang et al., 2024; Wang R. T et al., 2022; Zhu et al., 2023), encompassing cardioprotection, anti-inflammatory, antioxidative, and antineoplastic effects (Figure 1). This compound’s efficacy in mitigating cardiovascular pathologies (Bukarica et al., 2013) and its potential in oncological therapy underscore its significant therapeutic promise (Bukarica et al., 2013; Chen and Musa, 2021). The breadth of resveratrol’s bioactivity predicates the necessity of continued research to fully exploit its disease-preventive and therapeutic capacities.

Res has received a lot of attention from the scientific community, and it has become a focal point for the study of ferroptosis, an iron-dependent form of cell death that has been linked to a variety of diseases (Stockwell, 2022). The hallmark of ferroptosis is the buildup of iron and lipid peroxidation, which sets off a chain reaction. This reaction involves the exhaustion of glutathione (GSH) and a decrease in the effectiveness of glutathione peroxidase 4 (GPX4), leading to a rise in lipid peroxides and an increase in reactive oxygen species (ROS) levels (Dai et al., 2024; Liu YE et al., 2023). Such biochemical dynamics not only precipitate ferroptosis but also perturb amino acid metabolism and inflict mitochondrial damage (Jiang et al., 2021; Zheng and Conrad, 2020). The implications of ferroptosis extend beyond its biochemical pathways; it is intricately linked to the pathogenesis and progression of a variety of diseases, including cardiovascular disorders and cancer (Nie et al., 2024). The regulation of ferroptosis, therefore, unveils novel therapeutic avenues, enriching our arsenal against these conditions and enhancing our comprehension of cellular mortality mechanisms.

Res modulates ferroptosis by regulating lipid peroxidation and iron homeostasis and shows considerable promise in cardiovascular and oncological therapeutic areas (Cheng et al., 2020; Rauf et al., 2018; Ren et al., 2021; Xia et al., 2017; Zordoky et al., 2015). Its efficacy is particularly evident in its interaction with Toll-like receptor 4 (TLR4), showing promise in managing coronary heart disease and gastric cancer (Huo et al., 2019; Li MY et al., 2023). The influence of Res on ferroptosis is characterized by a dualistic nature: it can precipitate cell death by downregulating the expression of GPX4 and SLC7A11, offering a novel approach to cancer therapy (Shan et al., 2023); conversely, it can confer cellular protection by upregulating these genes, thereby inhibiting ferroptosis.

**FIGURE 2:**
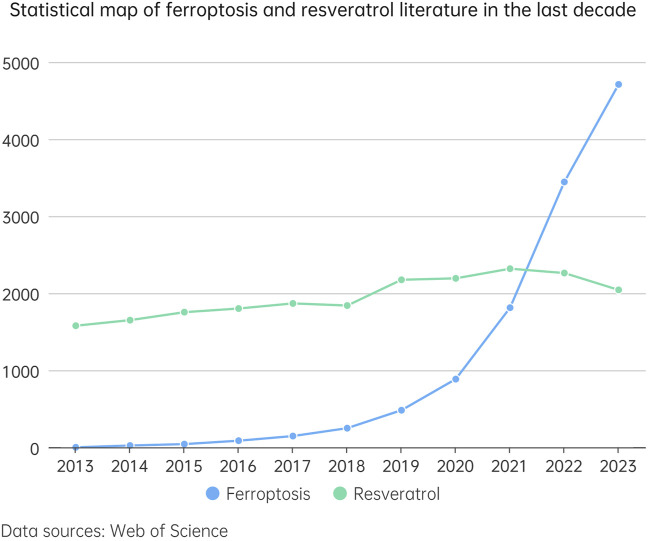
This line chart illustrates the trend in the number of studies related to ferroptosis and resveratrol over the past decade (2013–2023). In the chart, the blue dashed line represents the number of studies concerning ferroptosis, while the green dashed line indicates the number of studies on resveratrol. By comparing the trends of these two dashed lines, we can gain an intuitive understanding of the research interest and development trends in these two fields over the last decade.

**FIGURE 3:**
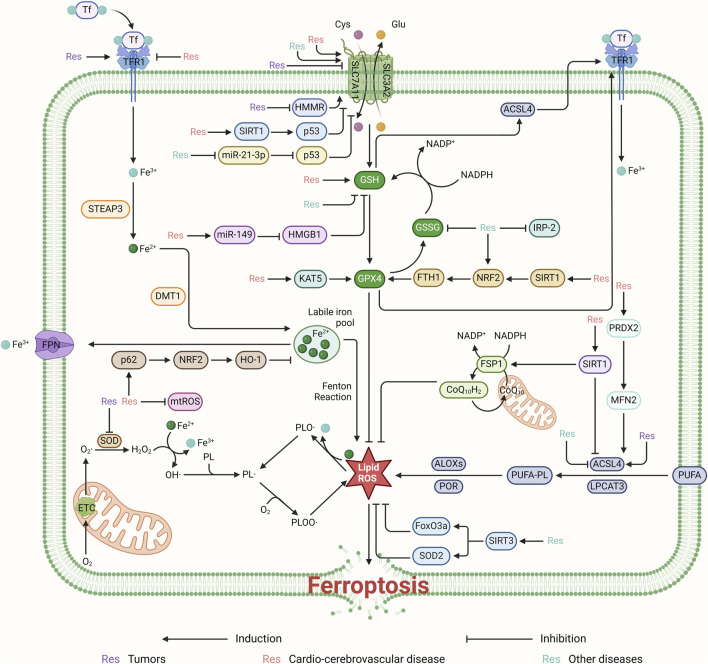
Resveratrol regulates ROS production and thus ferroptosis mainly through the system Xc^−^-GSH-GPX4 pathway. It can regulate ferroptosis for the treatment of cardiovascular diseases by activating the SIRT1/p53 and miR-149/HMGB1 signaling pathways, targeting SLC7A11 and GSH, respectively. Furthermore, resveratrol promotes ferroptosis inhibition by suppressing HMMR and the miR-21-3p/p53 signaling pathway, thereby affecting the system Xc^−^-GSH-GPX4 pathway. By upregulating the KAT5 and SIRT1/NRF2/FTH1 pathways, resveratrol can also target and enhance the expression of GPX4, inhibiting ROS production and ferroptosis. This contributes to the treatment of cardiovascular and cerebrovascular diseases. Resveratrol inhibits ROS production by targeting mtROS and by promoting the p62/NRF2/HO-1 pathway. Additionally, it regulates ROS production by activating the PRDX2/MFN2 pathway and upregulating SIRT1’s targeting of ACSL4. Resveratrol contributes to cellular protection by inhibiting ROS and ferroptosis through the activation of SIRT3, which in turn boosts the levels of FoxO3a and SOD2.

Res has garnered significant interest for its role in modulating ferroptosis and its therapeutic potential across a spectrum of diseases (Figure 2). Despite the burgeoning body of research in this domain, a comprehensive analysis elucidating resveratrol’s mechanism of action remains conspicuously absent. This review aims to fill this knowledge gap by organizing and discussing how Res affects ferroptosis and disease remission (Figure 3). By delineating its mechanistic pathways, this synthesis aims not only to keep the scientific community abreast of current developments but also to underpin the theoretical foundations for resveratrol’s clinical application in managing ferroptosis-related disorders.

## 2 Unraveling ferroptosis: mechanistic insights and resveratrol's impact

Ferroptosis, a recently identified mode of cell death, has rapidly ascended to prominence within the realm of biomedical research. Emerging studies have elucidated its intricate biological underpinnings and underscored its pivotal contribution to the pathogenesis of a diverse array of diseases. Through a detailed examination of the latest findings, we endeavor to provide a comprehensive overview that not only informs but also advances the potential for therapeutic interventions targeting ferroptosis.

### 2.1 Advance in understanding ferroptosis

Iron accumulation within cells is a critical factor in ferroptosis. After entering the cell via the transferrin receptor TFR1, iron is reduced to Fe^2+^ in the endoplasmic reticulum and then released into the cytoplasm via the SLC11A2 transporter. During ferroptosis, ROS react with polyunsaturated fatty acids (PUFAs), initiating lipid peroxidation. The accumulation of ROS is a key factor in cell death, and the inhibition of antioxidants such as GPX4 leads to ROS accumulation and lipid peroxidation, thereby inducing ferroptosis. Thus, the accumulation of iron, ROS, and markers like GPX4 all contribute to the onset of ferroptosis (Pierzynowska et al., 2021).

Ferroptosis, is increasingly recognized for its pivotal role in the biomedical sciences. Recent advances have significantly expanded our comprehension of its biological underpinnings, paving the way for novel therapeutic approaches across a spectrum of diseases. In terms of cellular morphology, ferroptosis is characterized by increased mitochondrial membrane density, reduction or disappearance of mitochondrial cristae, and rupture of the outer mitochondrial membrane (Zhao et al., 2023). At the molecular level, the identification of critical regulators such as Kinofen has shed light on the intricate control mechanisms governing ferroptosis, offering promising directions for clinical translation (Shen et al., 2023). In oncology, the discovery of GINS4’s suppressive impact on lung adenocarcinoma heralds a new therapeutic target (Chen L et al., 2023; Li et al., 2021; Li MT et al., 2022). Similarly, advancements in hepatology have been marked by the identification of ME1, which provides fresh insights into liver disease management (Fang et al., 2023). Cardiovascular research has underscored the importance of ALOX15 and its metabolites in ferroptosis, suggesting innovative treatment modalities for heart diseases (Cai et al., 2023; Sun et al., 2020). The elucidation of ferroptosis’s role in hypertensive nephrosclerosis also furnishes a novel theoretical foundation for addressing hypertension and its sequelae (Du et al., 2023). Moreover, investigations into conditions such as ischemic heart failure, degenerative bone diseases, and cognitive impairments have yielded significant breakthroughs, offering new perspectives and therapeutic avenues (Cheng et al., 2023; Ru et al., 2023; Yuan et al., 2023). These collective findings not only deepen our understanding of ferroptosis but also establish a robust basis for the development of future treatment strategies, underscoring the mechanism’s broad implications for health and disease.

/Recently, advancements in the study of ferroptosis have elucidated the fundamental roles of specific proteins and pathways, notably GPX4, P53, the Xc^−^-GSH-GPX4 and Xc-NAD(P)H/FSP1/CoQ10 axis, in the regulation of this cell death mechanism (Zheng and Conrad, 2020; Lee et al., 2021). The suppression of ferroptosis by GPX4, in particular, has emerged as a pivotal process (Li et al., 2020; Liu Y et al., 2023), with its modulation revealing therapeutic promise in the management of conditions such as heart failure (Zhang et al., 2023) and colorectal cancer (Zhang et al., 2022). In addition, PUFAs undergo lipid peroxidation affecting ferroptosis after entering the cell membrane via ACLS4 and LPCAT3 (Liu et al., 2022a). Moreover, DNA oxidative damage events can activate the cell’s DNA damage response, further triggering various cell death pathways, including ferroptosis. In the process of ferroptosis, cathepsin B (CTSB) plays a crucial role by translocating from lysosomes to the nucleus, causing DNA damage, a process that activates the STING1-dependent ferroptotic pathway. Furthermore, high mobility group box 1 (HMGB1) also functions in ferroptosis by activating the RAS-JNK/p38 signaling pathway, promoting DNA oxidative damage induced by erastin (Chen et al., 2021). These discoveries not only augment our comprehension of ferroptosis but also herald new therapeutic targets and strategies for combating related diseases, underscoring the intricate interplay between molecular pathways and their potential clinical applications.

### 2.2 Resveratrol: a key modulator of ferroptosis

Res has emerged as a pivotal regulator of ferroptosis. This compound exerts a nuanced influence on the process by meticulously modulating iron uptake and metabolism (Wang H et al., 2022). Specifically, Res governs the activity of the transferrin receptor (TFRC) and the SLC7A11 channel, orchestrating the influx of iron ions into cells, safeguarding iron homeostasis, and mitigating the risk of iron overload (Shan et al., 2023). Res activates the Nrf2 signaling pathway, promoting the translocation of Nrf2 from the cytoplasm to the nucleus, thereby enhancing the expression of a series of antioxidant enzymes, including superoxide dismutase (SOD), glutathione S-transferases (GSTs), and GPX. These enzymes play an important role in improving the cell’s antioxidant capacity. In addition, Res can stimulate the synthesis of GSH, increasing the level of GSH within cells and further enhancing the cell’s defense against oxidative stress. In terms of tumor suppression, Res activates the p53 protein, promoting its accumulation in the nucleus, which not only helps to promote cell cycle arrest and apoptosis, inhibiting the growth of tumor cells, but also p53 can suppress the expression of SLC7A11, reducing the cell’s uptake of cystine, thereby decreasing the synthesis of intracellular glutathione and making cells more sensitive to ferroptosis, a newly discovered form of cell death. This provides a new mechanistic explanation for the use of Res in the prevention and treatment of cardiovascular and cerebrovascular diseases and cancer (Jiang et al., 2015).

Moreover, Res plays a critical role in mitigating oxidative stress by scavenging hydroxyl radicals and curbing the production of ROS, thus preserving the intracellular ROS equilibrium and averting ferroptosis triggered by oxidative damage. It also possesses the unique capacity to suppress excessive mitochondrial ROS production while potentiating ROS activity within cancer cells, thereby erecting a cellular defense against ferroptosis (Fu et al., 2021; Zhang et al., 2019). At the proteomic level, Res subtly regulates the expression of key proteins such as SOD, SIRT1, ACSL4, FSP1, FTH1, GPX4, TfR1, and SLC7A11, indirectly preventing ferroptosis by either promoting or inhibiting the expression of these genes. These multifaceted regulatory mechanisms collectively delineate a comprehensive strategy for cellular protection against ferroptosis, unveiling novel avenues and methodologies for the therapeutic intervention of associated pathologies.

## 3 Targeted ferroptosis: resveratrol's role in disease intervention

### 3.1 Resveratrol modulates ferroptosis: a strategy against cardiovascular and cerebrovascular diseases

Cardiovascular and cerebrovascular diseases prevalently afflict the middle-aged and elderly populations, presenting a formidable challenge to public health due to their high incidence and mortality rates (Tang and Chan, 2014; Townsend et al., 2019). Despite continuous advancements in therapeutic approaches, the quality of life for many patients remains compromised. Res emerges as a beacon of hope in the treatment landscape for conditions such as coronary heart disease, heart failure, cerebral hemorrhage, and brain injury, by targeting the ferroptosis pathway (Table 1). This intervention strategy not only holds the potential to mitigate the progression of these debilitating diseases but also to significantly enhance patient outcomes, thereby addressing a critical need in contemporary medical practice.

**TABLE 1:** Overview of Resveratrol’s mechanisms in managing cardiovascular and cerebrovascular conditions.

Disease	Model	Concentration of res	Discovery	Mechanism	Ferroptosis	Effects on biomarkers	Ref
Valvular atrial fibrillation	Atrial fibrillation model/Molecular docking model	HL-1 cells (50 μM)	Inhibited NADPH oxidase and ion channels to improve myocardial fibrosis	Docked with TGFBR1 and regulated TGFβ/Smad signaling	Inhibition	TGFBR1↓, vimentin ↓, α-SMA↓, type I collagen↓	Jiang et al. (2022a)
Heart failure	Heart failure model	Mice (35 μM)	Improved cardiac function/slow ferroptosis and fibrosis progression in heart failure	Regulated Sirt1/p53 pathway, and reduced SLC7A11 consumption	Inhibition	SIRT1↑, p53K382 acetylation level↓, SLC7A11 degradation↓, GSH↑, GPX4↑	Jiang et al. (2022b)
Myocardial ischemia-reperfusion injury	OGD/R model *in vitro*/Myocardial I/R model *in vivo*	H9c2 cells (10 μM); Rats (50 mg/kg)	Reduced the level of oxidative stress and Fe^2+^ content, reduced the expression of TfR1, increased the expression of FTH1 and GPX4, prevented and treated myocardial I/R injury	Regulated USP19/Beclin1-induced autophagy in H9c2 cells to reduce oxidative stress level and Fe^2+^ content	Inhibition	Fe^2+^↓, TfR1↓, FTH1↑, GPX4↑	Li T et al. (2022)
Myocardial Injury	MI rat model	H9c2 cells (50 μM); Rats (20 mg/day)	Reduced myocardial injury and fibrosis related to myocardial infarction in rats	Regulated KAT5/GPX4 pathway	Inhibition	Collagen 1↓, α-SMA↓, IL-6↓, IL-1β↓, GSH↑, MDA↓, lipid ROS↓, Fe^2+^↓, GPX4↑, SLC7A11↑	Liu et al. (2022a)
Endotoxemia	A mouse model of LPS-induced endotoxemia/A cell model of endotoxemia induced myocardial injury	Mice (50 mg/kg)	Improved myocardial cell injury and cardiac function in LPS-induced endotoxemia	Regulated the miR-149/HMGB1 axis	Inhibition	miR-149↑, HMGB1↓, GSH↑, LDH↑, Lipid ROS↑, lipid peroxidation↓, Iron accumulation↓	Wang RT et al. (2022)
Suppurative myocardial injury	LPS induced SMI model in mice	—	Considered to be the drug with the strongest prediction of key genes related to ferroptosis	Docked with the key FRG	Inhibition	\	Zou et al. (2023)
Sepsis-induced cardiomyopathy	A rat model of sepsis induced by CLP	Rats (10, 30 and 50 mg/kg); H9c2 cells (1, 10, 25, 75, and 100 μM)	Reduced myocardial injury caused by sepsis	Regulated Sirt1/Nrf2 signaling pathway	Inhibition	SIRT1↑, GPX4↑, ACSL4↑, MDA↓, 4-HNE↓, Fe^2+^↓	Zeng et al. (2023)
Azithromycin-induced cardiotoxicity	—	—	Improved the left ventricular function and Myocardial fibrosis	Inhibited the overproduction of ROS and Regulated the p62-NRF2/HO-1 pathway	Inhibition	mtROS↓, p62-NRF2/HO-1↑, NRF2↑	Yu et al. (2022)
Azithromycin-induced cardiotoxicity	DIC model	Mice (20 mg/kg/day)	Alleviated DOX-induced cardiac dysfunction	Regulated MAPK signaling pathway	Inhibition	GSH↑, Fe accumulation ↓, PTGS2↓, ACSL4↓, NCOA4↓, GPX4↑, ROS↓	Chen et al. (2024)
5-FU-induced cardiotoxicity	Mouse model of cardiotoxicity	Mice (1,2,4 mg/kg); H9c2 cells (12.5, 25, 50 μM)	Alleviated 5-FU-induced cardiotoxicity	Upregulated GPX4	Inhibition	CK↓, LDH↓, MDA↓, Fe^2+^↓, GSH↑, GPX4↑, FTH1↑, Nrf2↑, NQO1↑, TfR↓, p53↓	Li DN et al. (2023)
Diabetic cardiac microvascular dysfunction	db/db mouse model	Mice (25 mg/kg/d)	Reduced the damage of vascular structure	Regulated PRDX2-MFN2-ACSL4 pathway	Inhibition	PRDX2↑, lipid peroxidation↓, MFN2↑, ACSL4↑	Chen L et al. (2023)
Diabetic cardiac microvascular dysfunction and cardiomyopathy	db/db mouse model	Mice (25 mg/kg/d)	Prevented the damage of endothelial cells in cardiac microvessels induced by glucolipotoxicity, and reduced the abnormal structure and function of microvessels in diabetic patients	Regulated PRDX2-MFN2-ACSL4 pathway	Inhibition	PRDX2↑, lipid peroxidation↓, MFN2↑, ACSL4↑	Liu (2023)
Cerebral ischemia-reperfusion injury	*In vitro* neuronal OGD/R/*in vivo* MCAO/R models	Neurons (0, 5, 10 and 20 μM); Rats (30 mg/kg)	Be similar to ferritin-1	Inhibited GPX4 and ferritin degradation, regulated Sirt1-Nrf2 pathway	Inhibition	GSH↑, ROS↓, ACSL4↓, ferritin↑, GPX4↑	Zhu et al. (2022)
Hypoxic-ischemic brain damage	Neonatal rat model of HIBI	Mice (25 μg/3 μL)	Protected the nerve, improved HIBI-induced brain atrophy/injury, and treated HIBI-induced learning and memory impairment	Regulated SIRT1/Nrf2/GPx4 signaling pathway	Inhibition	SIRT1↑, Nrf2↑, GPX4↑	Li MT et al. (2022)
Subarachnoid hemorrhage	*In vivo/in vitro* SAH model	Mice experiment (30 mg/kg); Mice experiment (50 μM)	Reduced the level of lipid peroxidation by upregulation of GPX4 and FSP1 expression	Regulated SIRT1 signaling pathway	Inhibition	SIRT1↑, ACSL4↓, GPX4↑, FSP1↑,	Yuan et al. (2022)
Cerebral hemorrhage	ICH mouse model/SD rat model	SD Rats (5 mg/kg)	Found the Res-NPs is a safer and more effective method for the treatment of cerebral hemorrhage injury	—	Inhibition	—	Mo et al. (2021)

#### 3.1.1 Cardiovascular diseases

In recent years, the role of ferroptosis in the pathogenesis of cardiovascular diseases has been gradually emphasized. Studies have shown that ferroptosis is associated with a variety of cardiac diseases including myocardial infarction, myocardial ischemia-reperfusion injury, cardiac fibrosis, atrial fibrillation, and cardiotoxicity. Res has been shown to have cardiovascular protective effects in several studies, especially in inhibiting ferroptosis by regulating the TLR4/NF-κB signaling pathway, TGFBR1 expression, and the Sirt1/p53/SLC7A11 pathway.

##### 3.1.1.1 Effects of resveratrol on simple cardiovascular disease: alleviation of myocardial infarction and heart failure

Myocardial infarction, as a serious manifestation of coronary artery disease, essentially refers to the necrosis of the myocardium due to ischemia, which is a severe type of acute coronary syndrome (Lindsey et al., 2018). In recent years, the role of ferroptosis in the pathogenesis of myocardial infarction has gradually received attention. A study by Jiang et al. found that ferroptosis is involved in the pathogenesis of myocardial infarction (Jiang et al., 2022a). Building on this foundation, Res has emerged as a preventative measure against myocardial ischemia-reperfusion injury by mitigating ferroptosis via the TLR4/NF-κB pathway, a critical link in ferroptosis and immune response dynamics. Res can stably bind to TLR4 with a minimum binding free energy of −6.2 kcal/mol and act synergistically to inhibit the growth of neuroendocrine tumors, thereby preventing myocardial injury after myocardial infarction. It was shown that depletion of TLR4 can significantly improve iron and lipid peroxidation in ischemia-reperfusion rat hearts and reduce the risk of cardiac remodeling and myocardial death.

Transitioning from the molecular to the clinical perspective, coronary heart disease and atrial fibrillation share an intimate link, often manifesting with similar clinical symptoms such as palpitations, chest pain and so on. Jiang et al.'s experiments further corroborated that TGFBR1, a pleiotropic cytokine, is not only participates in the immune responses but also plays a crucial role in tissue fibrosis induced by ferroptosis (Jiang et al., 2022b). Notably, both curcumin and Res can modulate TGFBR1 expression, with Res exhibiting an optimal binding free energy of 8.5 kcal/mol with TGFBR1. At this binding affinity, Res can effectively bind to its target site, reducing myocardial fibrosis and consequently ameliorating atrial fibrillation symptoms. This segues into the broader implications of resveratrol’s action, where in patients with coronary artery disease, cardiac vascular lesions further exacerbate myocardial ischemia and injury, significantly increasing the risk of heart failure. Zhang et al. demonstrated that Res acts as a natural activator of Sirt1, inhibiting ferroptosis through the Sirt1/p53/SLC7A11 pathway (Zhang et al., 2023). An ATP assay kit determined 35 μM as the optimal Res concentration for treatment. An aortic constriction surgery-induced heart failure model was established, and *in vivo* and *in vitro* experiments confirmed resveratrol’s ability to inhibit ferroptosis in cardiomyocytes via the Sirt1/Xc^−^ pathway. In patients with early-stage heart failure, Res effectively inhibited ferroptosis and enhanced cardiac function by modulating Sirt1 expression. Furthermore, long-term Res treatment significantly reduced the level of fat oxidation, increased GSH and SOD expression in cardiac tissues, and upregulated GPX4 and SLC7A11 expression in cardiomyocytes, decelerating the ferroptosis process and preserving left ventricular function.

Building on the understanding of myocardial ischemia-reperfusion injury, Li et al.'s study provides a deeper dive into the cellular mechanisms at play (Li T et al., 2022). Using the oxygen-glucose deprivation/reperfusion (OGD/R) model, it was found that OGD/R exposure led to a significant reduction in GPX4 and FTH1 mRNA and protein levels, along with an increase in TfR1 and LC3 levels and a decrease in p62 levels. And Res was found to improve cell viability as assessed by CCK-8. Subsequent experiments using 10 μM Res revealed that Res ameliorated OGD/R-induced oxidative stress by decreasing malondialdehyde (MDA) levels while enhancing SOD activity. Regarding its inhibitory effect on ferroptosis, Res primarily achieved this by increasing GPX4 and FTH1 protein expression. Additionally, after Res treatment, the protein levels of autophagy markers such as Beclin-1, NCOA4, and LC3 were significantly reduced in H9c2 cells. An I/R rat model was also established, and it was found that Res could intervene to downregulate the expression of USP19 and Beclin-1 while upregulating the expression of GPX4 and FTH1, and effectively reducing oxidative stress levels and Fe^2+^ content in cells and tissues. In conclusion, apoptosis is triggered by OGD/R-induced ferroptosis and excessive autophagy activation in H9c2 cells, whereas Res could prevent myocardial ischemia/reperfusion injury by regulating USP19/Beclin-1-induced autophagy in H9c2 cells and reducing oxidative stress levels and Fe^2+^ content, thereby inhibiting ferroptosis.

Transitioning from the context of ischemia-reperfusion injury to myocardial infarction, Liu et al.'s research further validates the protective role of Res (Liu et al., 2022b). Demonstrated that Res could inhibit ferroptosis and attenuate myocardial injury and fibrosis caused by myocardial infarction in rats by targeting the KAT5/GPX4 pathway, further corroborating the positive role of Res in treating myocardial infarction. They established a rat model of MI and injected the treatment group with 20 mg/day of Res, while the control group received an equal volume of PBS instead of Res. The study demonstrated that Res reduced the expression of collagen 1 and α-SMA, mitigated the increased levels of GSH, MDA, ROS, and Fe^2+^ induced by MI, and restored the expression of GPX4 and SLC7A11 that were decreased in MI rats. Additional *in vitro* experiments confirmed that Res could inhibit ferroptosis in cardiomyocytes induced by oxygen-glucose deprivation (OGD). Mechanistic studies revealed that Res rescued GPX4 expression levels and attenuated OGD-induced myocardial injury and ferroptosis by modulating KAT5 overexpression. Deletion of either KAT5 or GPX4 negated the cardioprotective effects of Res against OGD-induced injury in cardiomyocytes.

##### 3.1.1.2 Resveratrol: a potent ferritinase inhibitor in infectious cardiovascular disease

Transitioning from the broader implications of ferroptosis in cardiovascular diseases to its specific impact on conditions triggered by infectious agents, the exploration of endotoxemia and sepsis becomes particularly pertinent. The first is endotoxemia, which is a pathophysiological condition caused by the release of large amounts of endotoxin into the bloodstream by bacteria in the bloodstream or bacteria in the lesion, or by the infusion of endotoxin-contaminated fluid, often accompanied by myocardial injury.

In this context, a study by Wang et al. unveiled the protective effect of Res against myocardial injury in this condition (Wang XL et al., 2022). Ninety mice were randomly divided into nine groups, with a Res concentration of 50 mg/kg. The experimental results revealed that Res could inhibit the ferroptosis pathway by upregulating miR-149 expression and downregulating HMGB1 expression, thereby effectively treating endotoxemia-triggered myocardial injury. After treatment, cell viability was enhanced, iron output was reduced, GSH levels were increased, and both lipid ROS generation and lipid peroxidation were alleviated. These demonstrated the potential of Res in the treatment of myocardial injury caused by endotoxemia. Furthermore, Zou et al. showed that the prevalent disruption of ferroptosis-related genes (FRGs) in the septic cardiac transcriptome suggests the pivotal role of ferroptosis in this pathologic process (Zou et al., 2023). While Res, as the most promising drug target-related drug with a composite score of 2975802, can form a stable complex with these key FRGs. Res can dock with all the key FRGs, especially PTEN and RELA. The interaction energy with -CDOCKER was 38.57 kcal/mol and 33.56 kcal/mol, respectively. This suggests that it may be a potential drug for the treatment of septic myocardial injury by modulating ferroptosis.

Shifting focus to another critical condition associated with infectious diseases, septic cardiomyopathy, reveals another dimension of resveratrol’s therapeutic potential. For septic cardiomyopathy, Res, as a natural agonist of Sirt1, has a protective effect on it. Experiments by Zeng et al. demonstrated that Res could inhibit ferroptosis by upregulating the Sirt1/Nrf2 signaling pathway, that is, inhibiting ferroptosis by activating Sirt1 and Nrf2, effectively preventing sepsis-induced cardiac dysfunction and reducing the resulting cardiac insufficiency (Zeng et al., 2023). Mitochondrial dysfunction, a key factor in septic cardiomyopathy and an important indicator of ferroptosis, was also ameliorated by Res treatment. Res, a Sirt1 agonist, upregulated Sirt1, which induced Nrf2 levels in SIC rats. A concentration of 25 μM Res was evaluated to have the greatest therapeutic effect and was used for all subsequent experiments. After treatment with Res, lipid peroxidation was reduced, the expression of ACSL4 was inhibited, and the level of GPX4 was increased, and the degree of increase was proportional to the dose of Res, so that their content returned to stability. Furthermore, it reduced MDA and 4-HNE levels and lowered ferrous ion levels, thereby alleviating ferroptosis and CLP-induced tissue damage.

##### 3.1.1.3 Resveratrol as a protective agent: attenuating drug-induced cardiotoxicity

Moving from the general discussion on cardiotoxicity to specific instances, it's crucial to understand the multifaceted nature of drug-induced heart diseases. Cardiotoxicity is an important clinical problem that refers to the toxic effects of a drug on the myocardium or the cardiac electrical conduction system, leading to functional damage or structural abnormalities of the heart. Through *in vivo* and *in vitro* studies, Yu et al. treated H9c2 cells with 10 μM Fer-1 and then conducted experiments with Res with a concentration of 20 μM, revealed a key pathway for doxorubicin (DOX) to cause cardiotoxicity: iron-mediated oxygen free radicals to induce oxidative stress in cardiomyocytes (Yu et al., 2022). This finding confirms the central role of ferroptosis in DOX cardiotoxicity. Experimentally, DOX reduced the activity of H9c2 cells, leading to iron accumulation and lipid peroxidation. Whereas the intervention of Res effectively inhibited the overproduction of mitochondrial ROS and reversed these deleterious effects through the regulation of the p62-Nrf2/HO-1 pathway. Moreover, Res, as an activator of p62, demonstrated preventive and protective effects against DOX cardiotoxicity in a mouse model.

Building upon these findings, Chen et al. delved deeper into the protective mechanisms of Res against cardiotoxic agents (Chen et al., 2024). They selected a 20 μM concentration of Res for an in-depth study by establishing a DIC model and using the CCK-8 assay. The experimental results showed that Dox-induced reduction in cell viability and lactate dehydrogenase (LDH) release were significantly alleviated by Res treatment, which improved the iron accumulation phenomenon, reduced GSH depletion, and inhibited the expression of iron-death-related proteins, such as PTGS2, ACSL4, and NCOA4. Additionally, Res upregulated GPX4 levels and efficiently alleviated the generation of ROS and the lipid peroxidation process. In mechanistic studies, the research team investigated the mitogen-activated protein kinase (MAPK) subgroups p38, ERK, and c-Jun N-terminal kinase (JNK) in both *in vivo* and *in vitro* experiments. The findings indicate that resveratrol’s cardioprotective action may involve the inhibition of ferroptosis via the MAPK signaling pathway.

Further extending the scope of research to include another common chemotherapeutic agent, Li et al.'s study offers new insights into addressing the cardiotoxicity caused by 5-Fluorouracil (5-FU) (Li DN et al., 2023). They established a cardiotoxicity model through intraperitoneal injection of 5-FU (30 mg/kg), dividing it into five groups: the model group (saline), three Res dosage groups (low, medium, and high doses at 1, 2, 4 mg/kg), and the Fer-1 positive control group (2.5 mg/kg). The findings showed that the model group’s body weight significantly decreased compared to the control group, with the high-dose Res group showing a notable increase from 16.6 ± 0.40 to 23.3 ± 0.75 g. Lipid accumulation in the Res treatment groups also increased by factors of 2.17, 1.72, 1.30, and 0.97, respectively, compared to the control group. These results indicate that Res can mitigate 5-FU-induced lipid damage by regulating lipid oxidation and inhibit cardiac ferroptosis triggered by 5-FU via a GPX4-mediated mechanism. Specifically, the expression of GPX4 was significantly upregulated by Res, which effectively inhibited the ferroptosis process by reducing harmful hydroperoxides to harmless lipid alcohols under the combined action of GSH and GPX4. In the mouse model, Res also significantly improved left ventricular function, alleviated myofibrillar degeneration, and reduced mitochondrial damage, demonstrating its remarkable effect in lowering oxidative stress in the heart.

##### 3.1.1.4 Resveratrol: fighting cardiac microvascular complications in diabetes mellitus

Recent studies have also unveiled that Res and its analogs exert an ameliorative effect on diabetic cardiac microvascular disease by regulating ferroptosis. Diabetic cardiac microvascular injury is a unique and serious complication of diabetes. A study by Chen et al. revealed the antioxidant and cardiovascular protective efficacy of isoresveratrol, an analog of Res (Chen YQ et al., 2023). Experiments using ISO at a concentration of 10 μM revealed that PRDX2, as a downstream target of isoresveratrol, plays a pivotal role in the inhibition of oxidative stress, iron overload, and lipid peroxidation, thus mediating the inhibitory effect of isoresveratrol on ferroptosis. Additionally, overexpression of MFN2 prevented the mitochondrial ectopic localization of ACSL4, further inhibiting the ferroptosis process in mitochondria. Through these mechanisms, isoresveratrol attenuates iron-induced oxidative damage and lipid peroxidation in mitochondria via the PRDX2-MFN2-ACSL4 pathway, providing effective protection for the cardiac microvasculature in diabetic patients.

Based on the understanding of isoresveratrol’s protective effects, it's crucial to consider the broader implications of ferroptosis in diabetic complications. Type 2 diabetes mellitus (T2DM) not only increases the risk of coronary artery disease but may also lead to serious consequences such as cardiomyopathy and heart failure. Liu et al. synthesized numerous studies and pointed out that ferroptosis is a therapeutic target for cardiomyopathy in both diabetic and non-diabetic patients (Liu, 2023). In the db/db diabetes model, ferroptosis has a causative effect on cardiac microvascular endothelial dysfunction. Iron chelation or inhibition of ferroptosis can alleviate chemotherapy and ischemia/reperfusion-induced cardiomyopathy; and attenuation of endothelial lipid peroxidation can also inhibit ferroptosis, thus reducing the incidence of atherosclerosis (Blagov et al., 2023). Moreover, Res analogs, such as isorhapontigenin, exhibit antioxidant and anti-inflammatory properties and mitigate cardiac microvascular endothelial cell damage due to glycolipotoxicity through the PRDX2-MFN2-ACSL4 pathway, thereby improving microvascular structure and function in diabetic patients.

Taken together, Res and its derivatives demonstrate significant promise for treating myocardial infarction and associated cardiac conditions. They inhibit ferroptosis through multiple mechanisms, including modulation of key signaling pathways and gene expression, reduction of oxidative stress and ferric ion accumulation, reduction of lipid peroxidation, and enhancement of antioxidant system activity. In particular, the use of Res in diabetic cardiac microvascular disease, drug-induced cardiotoxicity, and restoration of cardiac function after myocardial infarction provides new strategies for the treatment of cardiac diseases. Future studies need to further explore the potential of resveratrol’s clinical applications and how it can be combined with other therapeutic approaches to achieve more effective treatment of cardiac diseases.

#### 3.1.2 Cerebrovascular diseases

Recent studies have highlighted resveratrol’s neuroprotective properties in cerebrovascular disease treatment, notably against oxygen-glucose deprivation/reoxygenation (OGD/R) injury. Zhu et al. found that OGD/R injury promotes ROS release, reducing GSH levels and causing iron accumulation (Zhu et al., 2022). Resveratrol’s preconditioning, involving exposure to varying concentrations (0–20 μM) for 24 h before OGD/R, demonstrated neuroprotective effects similar to the ferroptosis inhibitor ferritin-1 by inhibiting neuronal ferroptosis. Res was effective against OGD/R-induced ferroptosis and ferroptosis triggered by erastin and RSL3 in neurons. It also countered MCAO/R-induced ferroptosis in rats. The treatment reduced iron and ROS levels, inhibited ACSL4 expression, and enhanced GSH, ferritin, and GPX4 expression, mitigating mitochondrial damage. These actions collectively reduced neuronal ferroptosis, mirroring ferritin-1’s effects. Additionally, Res significantly reduced ferroptosis and mitochondrial damage in primary cortical neurons induced by erastin and RSL3 *in vitro*. It also showed promise *in vivo*, reducing degenerative neurons and the volume of cerebral ischemic injury and infarction by inhibiting ferroptosis after MCAO/R.

Beyond OGD/R injury’s impact, it's crucial to explore its relationship with other forms of brain damage. OGD/R injury is closely related to hypoxic-ischemic brain damage (HIBI) caused by perinatal asphyxia in terms of injury mechanism, pathophysiological process, and research methods. HIBI, a result of perinatal asphyxia, frequently culminates in cognitive deficits. Research conducted by Li et al. has unveiled a novel mechanism underlying HIBI: in a neonatal rat model, a transient increase in GPX4 levels was observed 6 hours post-HIBI, reaching a zenith at 24 hours, pinpointing ferroptosis as a pivotal event in HIBI pathogenesis (Li C et al., 2022). Ferrostatin-1 was identified as an effective inhibitor of this process, offering neuroprotection. Further investigations have elucidated that HIBI induces SIRT1 and Nrf2 expression while diminishing GPX4 levels, implicating the SIRT1/Nrf2/GPX4 signaling pathway in ferroptosis mediation. Significantly, Res, a SIRT1 agonist, markedly enhanced Sirt1, Nrf2, and GPX4 expression and concurrently decreased iron accumulation. This dual action not only thwarted ferroptosis via this pathway but also mitigated HIBI-induced neurological damage and memory impairments, presenting a promising avenue for therapeutic development.

Similarly, the exploration of HIBI’s mechanisms provides a segue into understanding the complexities of subarachnoid hemorrhage (SAH). Early brain injury (EBI) following SAH represents a multifaceted syndrome precipitated by various cerebrovascular conditions, predominantly attributed to intracranial aneurysm rupture. Research by Yuan et al. underscores the critical role of ferroptosis in EBI’s pathogenesis post-SAH (Yuan et al., 2022). Their *in vivo* and *in vitro* studies administered 30 mg/kg of Res and concentrations of 10 μM–100 μM of Res in DMEM, respectively, identifying 50 μM Res as the optimal dosage. Findings indicate that SIRT1, abundantly expressed in the brain, modulates oxidative and inflammatory pathways, thus offering neuroprotective benefits against EBI. Artificial augmentation of SIRT1 expression was observed to enhance GPX4 and FSP1 levels and significantly curtail lipid peroxidation, mitigating ferroptosis and attenuating EBI impacts. Consequently, SIRT1 emerges as a promising therapeutic target for SAH, with Res, as a SIRT1 activator, demonstrating significant potential in EBI treatment following SAH.

Intracerebral hemorrhage (ICH), characterized as a spontaneous hemorrhagic event within the brain parenchyma, primarily stems from cerebrovascular abnormalities. In this context, Res has been identified for its neuroprotective capabilities across various neuropathological conditions. A pivotal study by Mo et al. has advanced this narrative by employing polymer nanoparticles to encapsulate Res, creating spherical Res nanoparticles (Res-NPs) with an average diameter of 297.57 ± 7.07 nm (Mo et al., 2021). These Res-NPs demonstrated an enhanced capacity to maintain elevated Res levels. Experimental evaluations revealed that Res-NPs effectively mitigated erastin-induced ROS generation in HT22 cells and forestalled ferroptosis. Furthermore, in ICH mouse models, Nissl and Fluoro-Jade B staining corroborated the significant neuroprotective effect of Res-NPs, while Prussian blue staining evidenced their efficacy in reducing iron accumulation associated with cerebral hemorrhage. These insights herald innovative approaches and methodologies for ICH management, spotlighting the therapeutic potential of nanotechnology-enhanced Res delivery systems.

### 3.2 Regulation of ferroptosis by resveratrol: potential role in tumor suppression

Recently, Res, known for its diverse biological activities, is increasingly recognized for its potential to trigger ferroptosis in cancer cells (Ko et al., 2017; Zhang and Xie, 2024). This intersection of ferroptosis and Res opens promising avenues for cancer therapy, suggesting a paradigm shift towards exploiting cell death mechanisms for therapeutic advantage.

Resveratrol’s antineoplastic effects have been observed across a spectrum of cancers, highlighting its versatility as a therapeutic agent. In colorectal cancer, an *in vivo* study using a 10 mg/kg dosage revealed resveratrol’s ability to significantly reduce cell proliferation (Zhang et al., 2022). This effect is attributed to increased ROS and lipid peroxidation, activating the ROS-dependent ferroptosis pathway and downregulating SLC7A11 and GPX4, which are crucial for ferroptosis induction. In addition, pretreatment with ferrostatin-1 notably decreased MDA levels and downregulated SLC7A11 and GPX4. For lung squamous cell carcinoma (LUSC), Res demonstrated a dose-dependent reduction in H520 cell viability, with a 50 μM concentration inducing ferroptosis by modulating key protein expressions and enhancing immunomodulatory cytokines (TNF-α, IFN-γ, IL-12, IL-2) (Shan et al., 2023). This dual action improved the tumor immune microenvironment and increased CD8^+^ T cell toxicity, presenting a novel approach in LUSC therapy. Resveratrol’s therapeutic efficacy extends to melanoma (Synowiec-Wojtarowicz et al., 2023), where it disrupts redox homeostasis to trigger ferroptosis, head and neck cancer (Lee et al., 2020), through promoting epithelial-mesenchymal transition (EMT) reprogramming for ferroptosis, and gastric cancer (Li MY et al., 2023), by inhibiting inflammatory tumor cell proliferation via the TLR4 receptor. These findings illustrate resveratrol’s versatility in cancer treatment, capable of engaging multiple signaling pathways and enhancing the body’s immune response, thereby amplifying its therapeutic impact.

### 3.3 Broadening the therapeutic scope: resveratrol in various diseases

Beyond its pivotal contributions to cardiovascular and oncological therapies, recent investigations have illuminated resveratrol’s broader therapeutic spectrum across diverse diseases via the modulation of specific signaling pathways, with a particular emphasis on those associated with ferroptosis (Ma et al., 2024; Xu et al., 2018).

Res has also become a therapeutic drug suitable for addressing some inflammatory problems, including spinal cord injury (SCI), high-intensity exercise-induced inflammation, and diabetic periodontitis. In Ni et al.'s spinal cord injury study, Res was shown to reduce lipid peroxidation and iron accumulation through activation of the NRF2/GPX4 pathway, thereby preventing ferroptosis to further promote neurological and motor recovery in a mouse model (Ni et al., 2023). Additionally, Res has proven beneficial in mitigating exercise-induced gastrointestinal syndrome (EIGS) and related colon injuries, which are linked to high-intensity exercise (Xu Z et al., 2023). Res reduces inflammation and ferritin deposition in the colon through activation of the Nrf2/FTH1/GPX4 pathway, which results in increased FTH1 expression, improved iron sequestration, and GPX4 activity. This multifaceted approach notably decreased levels of harmful ferrous and ferric ions, hydrogen peroxide, and MDA, while boosting GSH concentration, catalase (CAT) activity, and the expression of SLC7A11 and GPX4 proteins. These findings not only highlight resveratrol’s capacity to address EIGS but also broaden its therapeutic potential across various medical conditions, confirming its role as a multifunctional therapeutic agent. Furthermore, diabetic periodontitis is a complex disease exacerbated by the coexistence of periodontitis and diabetes, further illustrating the versatility of Res. A study simulating diabetic periodontitis *in vitro* demonstrated that 6.25 μg/mL of Res counteracted the condition through disruption of the Xc^−^ system (Li Y et al., 2023). This suggests its effectiveness in treating ferroptosis of alveolar bone osteoblasts in diabetic periodontitis. In addition, Res has therapeutic efficacy in the treatment of intestinal ischemia-reperfusion injury and endometriosis.Wang et al. found that Res reduced ROS levels by activating the SIRT3/FoxO3a pathway, while upregulating the antioxidant genes SOD2 and catalase (Wang XJ et al., 2023). This dual action effectively inhibits ferritin deposition and contributes to the recovery of intestinal ischemia-reperfusion injury. In the context of endometriosis, a study by Zou et al. highlights that Res inhibits endometriosis by down-regulating miR-21-3p to modulate the p53/SLC7A11 pathway and inhibit cystine extraction, thereby promoting ferroptosis (Zou et al., 2024).

Furthermore, oxaliplatin-induced toxicity, such as neurotoxicity and ototoxicity, induces ferroptosis by increasing lipid peroxidation and ROS levels. Res acts by activating Nrf2, a key transcription factor, enhancing the expression of GSH and iron regulatory protein 2 (IRP-2), and mitigating GPX4 depletion caused by oxaliplatin (Xu K et al., 2023). This mechanism disrupts the ferroptosis pathway, presenting a new strategy to alleviate oxaliplatin’s adverse effects. Moreover, resveratrol’s protective effects extend to combating hepatotoxicity caused by deoxynivalenol (DON), a widespread mycotoxin. In experiments with 0.4 μM DON and 8 μM, Res activated the SLC7A11-GSH-GPX4 signaling pathway, significantly increasing the mRNA expression of GPX4, SLC7A11, NQO1, GCLC, and Nrf2 (Wang PJ et al., 2023). This upregulation not only prevents ferroptosis but also protects hepatocytes from DON-induced damage, as evidenced by reductions in GSH and total intracellular ROS levels. These findings underscore resveratrol’s comprehensive antitoxicity capabilities, further solidifying its position as a versatile therapeutic agent.

In total, Res has demonstrated its potential therapeutic effects across a range of diseases, particularly in the realm of cardiovascular health. As a multifaceted natural compound, it has shown promise in simple cardiovascular diseases by modulating key signaling pathways such as TLR4/NF-κB, Sirt1/p53/SLC7A11, Sirt1/Xc^−^, TfR1, and KAT5/GPX4, thereby exerting protective effects. In infectious cardiovascular diseases, Res mitigate inflammation and oxidative stress by targeting HMGB1, miR-149, GSH, FRGs, Sirt1/Nrf2, ACSL4, and GPX4, thus safeguarding the heart from infectious insults. Regarding drug-induced cardiotoxicity, resveratrol’s ability to modulate p62-Nrf2/HO-1, PTGS2, ACSL4, NCOA4, MAPK, p38, ERK, JNK, and GSH/GPX4 pathways suggests its potential in reducing cardiac damage caused by medications. In the context of microvascular complications associated with diabetes, Res improve microvascular function by influencing the PRDX2-MFN2-ACSL4 pathway. Cerebrovascular diseases also benefit from resveratrol’s modulation of ACSL4, GSH, SIRT1/Nrf2/GPX4, and FSP1, potentially protecting the brain’s vasculature from injury. Lastly, in oncology, resveratrol’s impact on SLC7A11, GPX4, and TLR4 inhibit the growth and spread of tumor cells. Together, these studies highlight the therapeutic versatility of Res and its role in modulating key pathways to combat complex diseases, bringing new hope in the fight against cardiovascular and cerebrovascular diseases and tumors.

## 4 Resveratrol and ferroptosis: pioneering treatment strategies and future prospects

### 4.1 The dual role of resveratrol and emerging therapeutic insights

Ferroptosis, marked by lipid peroxidation and iron overload, is increasingly recognized for its therapeutic potential, with Res emerging as a key modulator (Figure 3) (Liang et al., 2022). Res can both inhibit and promote ferroptosis across various biological systems, showcasing its versatility in treating diseases like cardiovascular, cerebrovascular disorders, and cancer. In the context of cardiovascular and cerebrovascular diseases, Res uses multiple signaling pathways, including TLR4/NF-κB, to inhibit ferroptosis, and it regulates cellular autophagy, offering new treatments for myocardial ischemia-reperfusion injury. It also supports myocardial infarction recovery and heart failure treatment through the KAT5/GPX4 and Sirt1/p53 pathways, respectively. Additionally, Res mitigates myocardial injury from endotoxemia and sepsis by modulating gene expression, highlighting its potential in infectious cardiovascular conditions. In cancer therapy, resveratrol’s ability to regulate SLC7A11 and GPX4 expression promotes ferroptosis in cancer cells (Sun et al., 2023), opening new therapeutic avenues for colorectal cancer and lung squamous cell carcinoma. Its interaction with TLR4 allows for targeted treatment of coronary heart disease and gastric cancer, demonstrating resveratrol’s dual role in ferroptosis regulation based on disease and therapeutic needs.

Recent advancements in ferroptosis research have opened up new therapeutic avenues for various diseases. Ping’s team has discovered that an enzyme in the cholesterol synthesis pathway plays a key role in modulating ferroptosis sensitivity by regulating 7-dehydrocholesterol (7-DHC) levels, offering new insights for cancer and ischemic reperfusion-injury treatments (Li et al., 2024). Additionally, studies have identified that cell cycle blockade can suppress ferroptosis, presenting a novel approach to target cancers with slow cell cycle resistance. Du et al.'s research further contributes to this field by showing how Apurinic/apyrimidinic nucleic acid endonuclease 1 facilitates ferroptosis in hepatocellular carcinoma, suggesting a new strategy for hepatocellular carcinoma treatment (Du et al., 2024). Moreover, recent studies have found that inducing the GSH/GPX4 axis to inhibit ferroptosis can serve as a new approach to treating stroke. This discovery of the signaling pathway offers a novel perspective for the use of resveratrol in stroke therapy, based on the modulation of ferroptosis (Wang YM et al., 2023; Xu YF et al., 2023). These findings not only broaden the scope of ferroptosis-based therapies but also highlight the importance of targeting specific molecular mechanisms to develop effective treatments for complex diseases.

### 4.2 Challenges in resveratrol’s clinical application

Despite promising developments, the clinical use of Res faces challenges due to its poor bioavailability, attributed to rapid metabolism and the first-pass effect (Smoliga and Blanchard, 2014). Addressing this, researchers have explored molecular structure modifications and advanced drug delivery systems to enhance resveratrol’s therapeutic potential (Li CH et al., 2023; Summerlin et al., 2015; Walle, 2011). By developing Res analogs, such as pterostilbene, researchers have managed to retain resveratrol’s biological activity while significantly improving its bioavailability. Pterostilbene, in particular, shows enhanced lipid solubility and reduced metabolic rate, leading to higher accumulation in colon cancer cell models compared to Res. The increased area under the curve in various cell lines underscores its enhanced bioavailability and potential in colon cancer treatment, offering a promising direction for resveratrol’s structural optimization and clinical utility. On the drug delivery front, encapsulating Res in nanocarriers like liposomes, polymer nanoparticles, and solid lipid nanoparticles has shown to protect it from rapid metabolism and enable targeted delivery (Saleem et al., 2022). This approach not only improves therapeutic efficacy and minimizes side effects but also allows for sustained and controlled drug release, thereby prolonging the drug’s presence in the body. Nanomedicine formulations address resveratrol’s poor water solubility and bring advantages such as prolonged circulation, biocompatibility, and degradability, broadening the scope for resveratrol’s clinical application.

### 4.3 Navigating the complexities of resveratrol: adverse effects, drug interactions, and research imperatives

The emergence of adverse side effects and potential drug interactions with Res consumption has become a growing concern (Zheng et al., 2012). Studies have shown that Res intake may cause side effects such as flatulence and nephrotoxicity (Howells et al., 2011; Poulsen et al., 2013). This highlights the urgent need for research aimed at reducing resveratrol’s toxicity and minimizing adverse reactions. Additionally, the use of Res alongside medications that have competitive binding sites is cautioned against due to the potential for reduced therapeutic efficacy and increased side effects, underscoring the complexity of its clinical use and the importance of thorough drug interaction studies. To optimize drug combinations and improve therapeutic outcomes, identifying compounds that may interact competitively with Res is crucial (Lagunas-Rangel, 2024). However, current research is limited by a lack of *in vivo* pharmacokinetic data in mouse models, restricting our understanding of resveratrol’s biological mechanisms and slowing progress. Moreover, while some experimental results, such as ferroptosis’s role in cardiomyopathy, have been partially confirmed *in vivo*, there is a lack of corroborative *in vitro* evidence. This discrepancy underscores the need for rigorous validation between *in vivo* and *in vitro* findings in future research. Additionally, the mechanisms and roles of key FRGs in molecular docking studies are not fully understood, indicating that further exploration could help translate these findings into clinical benefits.

## 5 Conclusion

Ferroptosis is an emerging focus in cell death research, closely associated with studies on Res. While advancements have been noted, our knowledge of its mechanisms and intercellular dynamics is still limited, highlighting the need for further research to delve into its complexities. The lack of definitive diagnostic markers for ferroptosis complicates its application in clinical diagnostics and therapy, making the identification of reliable markers and therapeutic targets crucial for translating ferroptosis research into effective healthcare solutions. Moreover, the key molecules and pathways that could leverage ferroptosis for disease treatment remain largely unidentified, emphasizing the importance of continued, in-depth research to discover new therapeutic targets and strategies. Despite resveratrol’s promising therapeutic potential, its side effects, potential drug interactions, and the current gaps in research call for careful exploration and sustained efforts to fully realize its clinical benefits.

## Figures and Tables

**Table:** 

**Res**	resveratrol
**GPX4**	glutathione peroxidase 4
**TLR4**	toll-like receptor 4
**ME1**	malic enzyme 1 gene
**TFRC**	transferrin receptor
**HO-1**	oxygenase-1
**ACSL4**	long-chain-fatty-acid-CoA ligase 4
**TfR1**	transferrin receptor protein 1
**FTH1**	ferritin heavy chain 1
**Sirt1**	silent information regulator 1
**GSH**	glutathione
**SOD**	superoxide dismutase
**OGD/R**	oxygen-glucose deprivation
**MDA**	malondialdehyde
**NCOA4**	nuclear receptor coactivator 4
**LC3**	microtubule-associated proteins light chain 3
**USP19**	ubiquitin specific peptidase 19 gene
**KAT5**	lysine acetyltransferase 5 gene
**PBS**	phosphate buffered saline
**α-SMA**	α-smooth muscle actin
**miR-149**	microRNA-149
**HMGB1**	high mobility group box 1 protein
**FRGs**	ferroptosis-related genes
**PTEN**	phosphatase and tensin homolog
**4-HNE**	4-hydroxynonenalal
**EBI**	early brain injury
**SAH**	subarachnoid hemorrhage
**CLP**	cecal ligation and puncture
**DOX**	doxorubicin
**5-FU**	5-fluorouracil
**LDH**	lactate dehydrogenase
**PTGS2**	prostaglandin-endoperoxide synthase 2
**ERK**	extracellular regulated protein kinases
**JNK**	c-Jun N-terminal kinase
**MAPK**	mitogen-activated protein kinase
**PRDX2**	peroxiredoxin 2 Gene
**MFN2**	mitofusin 2
**RSL3**	RAS-selective lethal 3
**FSP1**	ferroptosis suppressor protein 1
**IFN-γ**	interferon γ
**IL**	interleukin
**IRP-2**	iron regulatory protein-2
**NQO1**	NAD(P)H quinone dehydrogenase 1
**GCLC**	glutamate-cysteine ligase catalytic subunit
**SIRT3**	sirtuin 3
**TNF-α**	tumor necrosis factor-α
**FoxO3a**	forkhead box O3
**GSSG**	glutathione oxidized
**Ptgs2**	prostaglandin-endoperoxide synthase 2
**Chac1**	ChaC glutathione specific gamma-glutamylcyclotransferase 1 Gene
**7-DHC**	7-dehydrocholesterol
**Caco-2**	human intestinal epithelial cells
**OGD/R**	oxygen-glucose deprivation/reperfusion
**ROS**	reactive oxygen species
**HCT116**	HCT-116-eGFP
**TGFBR1**	fransforming growth factor, beta receptor 1
**MCAO/R**	middle cerebral artery occlusion and reperfusion

## References

[B1] Blagov A. V. Markin A. M. Bogatyreva A. I. Tolstik T. V. Sukhorukov V. N. Orekhov A. N. (2023). The role of macrophages in the pathogenesis of atherosclerosis. Cells 12, 522. 10.3390/cells12040522 36831189 PMC9954519

[B2] Bukarica L. G. Protic D. Kanjuh V. Heinle H. Novakovic R. Scepanovic R. (2013). Cardiovascular effects of resveratrol. Vojnosanit. Pregl. 70, 1145–1150. 10.2298/vsp120613012g 24450260

[B3] Cai W. B. Liu L. Shi X. L. Liu Y. A. Wang J. Fang X. (2023). Alox15/15-HpETE aggravates myocardial ischemia-reperfusion injury by promoting cardiomyocyte ferroptosis. Circulation 147, 1444–1460. 10.1161/CIRCULATIONAHA.122.060257 36987924

[B4] Chen L. Cai Q. D. Yang R. Wang H. Y. Ling H. L. Li T. S. (2023). GINS4 suppresses ferroptosis by antagonizing p53 acetylation with snail. P. Natl. Acad. Sci. U. S. A. 120, e2219585120. 10.1073/pnas.2219585120 PMC10104543 37018198

[B5] Chen L. Sun X. G. Wang Z. Chen M. He Y. X. Zhang H. (2024). Resveratrol protects against doxorubicin-induced cardiotoxicity by attenuating ferroptosis through modulating the MAPK signaling pathway. Toxicol. Appl. Pharm. 482, 116794. 10.1016/j.taap.2023.116794 38142782

[B6] Chen L. B. Musa A. E. (2021). Boosting immune system against cancer by resveratrol. Phytother. Res. 35, 5514–5526. 10.1002/ptr.7189 34101276

[B7] Chen X. Yu C. Kang R. Kroemer G. Tang D. (2021). Cellular degradation systems in ferroptosis. Cell. death. Differ. 28, 1135–1148. 10.1038/s41418-020-00728-1 33462411 PMC8027807

[B8] Chen Y. Q. Li S. Yin M. Li Y. F. Chen C. Zhang J. (2023). Isorhapontigenin attenuates cardiac microvascular injury in diabetes via the inhibition of mitochondria-associated ferroptosis through PRDX2-MFN2-ACSL4 pathways. Diabetes 72, 389–404. 10.2337/db22-0553 36367849

[B9] Cheng C. K. Luo J. Y. Lau C. W. Chen Z. Y. Tian X. Y. Huang Y. (2020). Pharmacological basis and new insights of resveratrol action in the cardiovascular system. Brit. J. Pharmacol. 177, 1258–1277. 10.1111/bph.14801 31347157 PMC7056472

[B10] Cheng Y. Gao Y. Li J. Rui T. Y. Li Q. Q. Chen H. (2023). TrkB agonist N-acetyl serotonin promotes functional recovery after traumatic brain injury by suppressing ferroptosis via the PI3K/Akt/Nrf2/Ferritin H pathway. Free. Radic. Bio. Med. 194, 184–198. 10.1016/j.freeradbiomed.2022.12.002 36493983

[B11] Dai E. Y. Chen X. Linkermann A. Jiang X. J. Kang R. Kagan V. E. (2024). A guideline on the molecular ecosystem regulating ferroptosis. Nat. Cell. Biol. 26, 1447–1457. 10.1038/s41556-024-01360-8 38424270 PMC11650678

[B12] Du X. Ma X. L. Tan Y. Shao F. Y. Li C. Zhao Y. (2023). B cell-derived anti-beta 2 glycoprotein I antibody mediates hyperhomocysteinemia-aggravated hypertensive glomerular lesions by triggering ferroptosis. Signal. Transduct. Target. 8, 103. 10.1038/s41392-023-01313-x PMC10008839 36907919

[B13] Du Y. Zhou Y. Yan X. Y. Pan F. Y. He L. F. Guo Z. G. (2024). APE1 inhibition enhances ferroptotic cell death and contributes to hepatocellular carcinoma therapy. Cell. death. Differ. 31, 431–446. 10.1038/s41418-024-01270-0 38418695 PMC11043431

[B14] Fang X. X. Zhang J. W. Li Y. Song Y. J. Yu Y. Y. Cai Z. X. (2023). Malic enzyme 1 as a novel anti-ferroptotic regulator in hepatic Ischemia/Reperfusion injury. Adv. Sci. 10, e2205436. 10.1002/advs.202205436 PMC10161122 36840630

[B15] Fu X. Li M. Tang C. L. Huang Z. Z. Najafi M. (2021). Targeting of cancer cell death mechanisms by resveratrol: a review. Apoptosis 26, 561–573. 10.1007/s10495-021-01689-7 34561763

[B16] Howells L. M. Berry D. P. Elliott P. J. Jacobson E. W. Hoffmann E. Hegarty B. (2011). Phase I randomized, double-blind pilot study of micronized resveratrol (SRT501) in patients with hepatic metastases-safety, pharmacokinetics, and pharmacodynamics. Cancer. Prev. Res. 4, 1419–1425. 10.1158/1940-6207.CAPR-11-0148 PMC3173869 21680702

[B17] Huo X. Y. Zhang T. Meng Q. F. Li C. X. You B. A. (2019). Resveratrol effects on a diabetic rat model with coronary heart disease. Med. Sci. Monit. 25, 540–546. 10.12659/MSM.910996 30658350 PMC6346847

[B18] Jiang F. Zeng Z. C. Zhou X. Tan M. L. Zhang W. W. Li M. Y. (2022a). Transcriptomic analysis uncovers immunogenic characteristics of ferroptosis for myocardial infarction and potential therapeutic prediction of Chinese herbs. Evid-Based. Compl. Alt. 2022, 4918343. 10.1155/2022/4918343 PMC9159883 35664944

[B19] Jiang F. Zhang W. W. Lu H. D. Tan M. L. Zeng Z. C. Song Y. Z. (2022b). Prediction of herbal medicines based on immune cell infiltration and immune- and ferroptosis-related gene expression levels to treat valvular atrial fibrillation. Front. Genet. 13, 886860. 10.3389/fgene.2022.886860 36246656 PMC9554472

[B20] Jiang L. Kon N. Li T. Y. Wang S. J. Su T. Hibshoosh H. (2015). Ferroptosis as a p53-mediated activity during tumour suppression. Nature 520, 57–62. 10.1038/nature14344 25799988 PMC4455927

[B21] Jiang X. J. Stockwell B. R. Conrad M. (2021). Ferroptosis: mechanisms, biology and role in disease. Nat. Rev. Mol. Cell. Bio. 22, 266–282. 10.1038/s41580-020-00324-8 33495651 PMC8142022

[B22] Ko J. H. Sethi G. Um J. Y. Shanmugam M. K. Arfuso F. Kumar A. P. (2017). The role of resveratrol in cancer therapy. Int. J. Mol. Sci. 18, 2589. 10.3390/ijms18122589 29194365 PMC5751192

[B23] Koushki M. Amiri-Dashatan N. Ahmadi N. Abbaszadeh H. A. Rezaei-Tavirani M. (2018). Resveratrol: a miraculous natural compound for diseases treatment. Food. Sci. Nutr. 6, 2473–2490. 10.1002/fsn3.855 30510749 PMC6261232

[B24] Lagunas-Rangel F. A. (2024). Prediction of resveratrol target proteins: a bioinformatics analysis. J. Biomol. Struct. Dyn. 42, 1088–1097. 10.1080/07391102.2023.2196698 37011009

[B25] Lee J. You J. H. Kim M. S. Roh J. L. (2020). Epigenetic reprogramming of epithelial-mesenchymal transition promotes ferroptosis of head and neck cancer. Redox. Biol. 37, 101697. 10.1016/j.redox.2020.101697 32896720 PMC7484553

[B26] Lee J. Y. Kim W. K. Bae K. H. Lee S. C. Lee E. W. (2021). Lipid metabolism and ferroptosis. Biol. (Basel). 10, 184. 10.3390/biology10030184 PMC8000263 33801564

[B27] Li D. N. Song C. Z. Zhang J. Zhao X. Y. (2023). Resveratrol alleviated 5-FU-induced cardiotoxicity by attenuating GPX4 dependent ferroptosis. J. Nutr. Biochem. 112, 109241. 10.1016/j.jnutbio.2022.109241 36442718

[B28] Li J. Cao F. Yin H. L. Huang Z. J. Lin Z. T. Mao N. (2020). Ferroptosis: past, present and future. Cell. death. Dis. 11, 88. 10.1038/s41419-020-2298-2 32015325 PMC6997353

[B29] Li M. T. Xie L. Jiang H. M. Huang Q. Tong R. S. Li X. (2022). Role of Licochalcone A in potential pharmacological therapy: a review. Front. Pharmacol. 13, 878776. 10.3389/fphar.2022.878776 35677438 PMC9168596

[B30] Li M. Y. Tao J. Qian R. Jiang F. Song Y. Z. Zeng Z. C. (2023). Development of alternative herbals remedy for gastric cancer based on transcriptomic analysis of immune infiltration and ferroptosis. Front. Genet. 14, 1086368. 10.3389/fgene.2023.1086368 36936437 PMC10020191

[B31] Li T. Tan Y. Ouyang S. He J. Liu L. L. (2022). Resveratrol protects against myocardial ischemia-reperfusion injury via attenuating ferroptosis. Gene 808, 145968. 10.1016/j.gene.2021.145968 34530090

[B32] Li X. Zhang C. T. Ma W. Xie X. Huang Q. (2021). Oridonin: a review of its pharmacology, pharmacokinetics and toxicity. Front. Pharmacol. 12, 645824. 10.3389/fphar.2021.645824 34295243 PMC8289702

[B33] Li Y. Huang Z. J. Pan S. F. Feng Y. H. He H. K. Cheng S. G. (2023). Resveratrol alleviates diabetic periodontitis-induced alveolar osteocyte ferroptosis possibly via regulation of SLC7A11/GPX4. Nutrients 15, 2115. 10.3390/nu15092115 37432277 PMC10181281

[B34] Li Y. X. Ran Q. Duan Q. H. Jin J. L. Wang Y. J. Yu L. (2024). 7-Dehydrocholesterol dictates ferroptosis sensitivity. Nature 626, 411–418. 10.1038/s41586-023-06983-9 38297130 PMC11298758

[B35] Liang D. G. Minikes A. M. Jiang X. J. (2022). Ferroptosis at the intersection of lipid metabolism and cellular signaling. Mol. Cell. 82, 2215–2227. 10.1016/j.molcel.2022.03.022 35390277 PMC9233073

[B36] Li C C. Wu Z. Y. Xue H. Gao Q. S. Zhang Y. H. Wang C. M. (2022). Ferroptosis contributes to hypoxic-ischemic brain injury in neonatal rats: role of the SIRT1/Nrf2/GPx4 signaling pathway. Cns. Neurosci. Ther. 28, 2268–2280. 10.1111/cns.13973 36184790 PMC9627393

[B37] Li Ch C. H. Wang Z. Lei H. Zhang D. (2023). Recent progress in nanotechnology-based drug carriers for resveratrol delivery. Drug. Deliv. 30, 2174206. 10.1080/10717544.2023.2174206 36852655 PMC9980162

[B38] Lindsey M. L. Bolli R. Canty J. M. Du X. J. Frangogiannis N. G. Frantz S. (2018). Guidelines for experimental models of myocardial ischemia and infarction. Am. J. Physiol-Heart. C 314, H812–H838. 10.1152/ajpheart.00335.2017 PMC5966768 29351451

[B39] Liu J. Kang R. Tang D. (2022a). Signaling pathways and defense mechanisms of ferroptosis. Febs. J. 289, 7038–7050. 10.1111/febs.16059 34092035

[B40] Liu J. Zhang M. M. Qin C. S. Wang Z. K. Chen J. H. Wang R. (2022b). Resveratrol attenuate myocardial injury by inhibiting ferroptosis *via* inducing KAT5/GPX4 in myocardial infarction. Front. Pharmacol. 13, 906073. 10.3389/fphar.2022.906073 35685642 PMC9171715

[B41] Liu Y. Wan Y. C. Jiang Y. Zhang L. Cheng W. J. (2023). GPX4: the hub of lipid oxidation, ferroptosis, disease and treatment. Bba-Rev. Cancer 1878, 188890. 10.1016/j.bbcan.2023.188890 37001616

[B42] Liu Y. E. Lu S. P. Wu L. L. Yang L. Yang L. X. Wang J. H. (2023). The diversified role of mitochondria in ferroptosis in cancer. Cell. death. Dis. 14, 519. 10.1038/s41419-023-06045-y 37580393 PMC10425449

[B43] Liu Z. Q. (2023). Cardiac microvascular dysfunction and cardiomyopathy in diabetes: is ferroptosis a therapeutic target? Diabetes 72, 313–315. 10.2337/dbi22-0036 36806606 PMC10090265

[B44] Ma Y. F. Qian Y. Chen Y. T. Ruan X. X. Peng X. Y. Sun Y. (2024). Resveratrol modulates the inflammatory response in hPDLSCs via the NRF2/HO-1 and NF-κB pathways and promotes osteogenic differentiation. J. Periodontal. Res. 59, 162–173. 10.1111/jre.13200 37905727

[B45] Mo Y. S. Duan L. N. Yang Y. N. Liu W. Zhang Y. Zhou L. G. (2021). Nanoparticles improved resveratrol brain delivery and its therapeutic efficacy against intracerebral hemorrhage. Nanoscale 13, 3827–3840. 10.1039/d0nr06249a 33565555

[B46] Mohammadipoor N. Shafiee F. Rostami A. Kahrizi M. S. Soleimanpour H. Ghodsi M. (2022). Resveratrol supplementation efficiently improves endothelial health: a systematic review and meta-analysis of randomized controlled trials. Phytother. Res. 36, 3529–3539. 10.1002/ptr.7562 35833325

[B47] Ni C. T. Ye Q. Mi X. D. Jiao D. Zhang S. S. Cheng R. D. (2023). Resveratrol inhibits ferroptosis via activating NRF2/GPX4 pathway in mice with spinal cord injury. Microsc. Res. Tech. 86, 1378–1390. 10.1002/jemt.24335 37129001

[B48] Nie A. Z. Shen C. Z. Zhou Z. Wang J. Sun B. Zhu C. S. (2024). Ferroptosis: potential opportunities for natural products in cancer therapy. Phytother. Res. 38, 1173–1190. 10.1002/ptr.8088 38116870

[B49] Pangeni R. Sahni J. K. Ali J. Sharma S. Baboota S. (2014). Resveratrol: review on therapeutic potential and recent advances in drug delivery. Expert. Opin. Drug. del. 11, 1285–1298. 10.1517/17425247.2014.919253 24830814

[B50] Pierzynowska K. Rintz E. Gaffke L. Węgrzyn G. (2021). Ferroptosis and its modulation by autophagy in light of the pathogenesis of lysosomal storage diseases. Cells 10, 365. 10.3390/cells10020365 33578654 PMC7916399

[B51] Poulsen M. M. Vestergaard P. F. Clasen B. F. Radko Y. Christensen L. P. Stodkilde-Jorgensen H. (2013). High-Dose resveratrol supplementation in obese men an investigator-initiated, randomized, placebo-controlled clinical trial of substrate metabolism, insulin sensitivity, and body composition. Diabetes 62, 1186–1195. 10.2337/db12-0975 23193181 PMC3609591

[B52] Rauf A. Imran M. Butt M. S. Nadeem M. Peters D. G. Mubarak M. S. (2018). Resveratrol as an anti-cancer agent: a review. Crit. Rev. Food. Sci. 58, 1428–1447. 10.1080/10408398.2016.1263597 28001084

[B53] Ren B. X. Kwah M. X. Y. Liu C. L. Ma Z. W. Shanmugam M. K. Ding L. W. (2021). Resveratrol for cancer therapy: challenges and future perspectives. Cancer. Lett. 515, 63–72. 10.1016/j.canlet.2021.05.001 34052324

[B54] Riccio B. V. F. Fonseca-Santos B. Ferrari P. C. Chorilli M. (2020). Characteristics, biological properties and analytical methods of trans-resveratrol: a review. Crit. Rev. Anal. Chem. 50, 339–358. 10.1080/10408347.2019.1637242 31353930

[B55] Ru Q. Li Y. S. Xie W. Q. Ding Y. L. Chen L. Xu G. D. (2023). Fighting age-related orthopedic diseases: focusing on ferroptosis. Bone. Res. 11, 12. 10.1038/s41413-023-00247-y 36854703 PMC9975200

[B56] Saleem Z. Rehman K. Akash M. S. H. (2022). Role of drug delivery system in improving the bioavailability of resveratrol. Curr. Pharm. Des. 28, 1632–1642. 10.2174/1381612828666220705113514 35792129

[B57] Shan G. Minchao K. Jizhao W. Rui Z. Guangjian Z. Jin Z. (2023). Resveratrol improves the cytotoxic effect of CD8^+^T cells in the tumor microenvironment by regulating HMMR/ferroptosis in lung squamous cell carcinoma. J. Pharm. Biomed. 229, 115346. 10.1016/j.jpba.2023.115346 37001272

[B58] Shen S. Y. Shen J. Luo Z. Wang F. D. Min J. X. (2023). Molecular mechanisms and clinical implications of the gold drug auranofin. Coord. Chem. Rev. 493, 215323. 10.1016/j.ccr.2023.215323

[B59] Smoliga J. M. Blanchard O. (2014). Enhancing the delivery of resveratrol in humans: if low bioavailability is the problem, what is the solution? Molecules 19, 17154–17172. 10.3390/molecules191117154 25347459 PMC6270951

[B60] Stockwell B. R. (2022). Ferroptosis turns 10: emerging mechanisms, physiological functions, and therapeutic applications. Cell 185, 2401–2421. 10.1016/j.cell.2022.06.003 35803244 PMC9273022

[B61] Summerlin N. Soo E. Thakur S. Qu Z. Jambhrunkar S. Popat A. (2015). Resveratrol nanoformulations: challenges and opportunities. Int. J. Pharm. 479 (2), 282–290. 10.1016/j.ijpharm.2015.01.003 25572692

[B62] Sun Q. Tao Q. Ming T. Tang S. Zhao H. Liu M. (2023). Berberine is a suppressor of Hedgehog signaling cascade in colorectal cancer. Phytomedicine 114, 154792. 10.1016/j.phymed.2023.154792 37028248

[B63] Sun Q. Xie L. Song J. Li X. (2020). Evodiamine: a review of its pharmacology, toxicity, pharmacokinetics and preparation researches. J. Ethnopharmacol. 262, 113164. 10.1016/j.jep.2020.113164 32738391

[B64] Synowiec-Wojtarowicz A. Krawczyk A. Kimsa-Dudek M. (2023). The effect of resveratrol and static magnetic field interactions on the oxidation-reduction parameters of melanoma malignant cells. Appl. Sci-Basel. 13, 8042. 10.3390/app13148042

[B65] Tang Y. L. Chan S. W. (2014). A Review of the pharmacological effects of piceatannol on cardiovascular diseases. Phytother. Res. 28, 1581–1588. 10.1002/ptr.5185 24919577

[B66] Townsend N. Wilson L. Bhatnagar P. Wickramasinghe K. Rayner M. Nichols M. (2019). Cardiovascular disease in europe: epidemiological update 2016. Eur. Heart. J. 40, 3232–3245. 10.1093/eurheartj/ehw334 PMC13480964 27523477

[B67] Vajdi M. Azar P. S. Mahmoodpoor A. Dashti F. Sanaie S. Chalmardi F. K. (2023). A comprehensive insight into the molecular and cellular mechanisms of action of resveratrol on complications of sepsis a systematic review. Phytother. Res. 37, 3780–3808. 10.1002/ptr.7917 37405908

[B68] Walle T. (2011). Bioavailability of resveratrol. Ann. N. Y. Acad. Sci. 1215, 9–15. 10.1111/j.1749-6632.2010.05842.x 21261636

[B69] Wang B. Jiang H. M. Qi L. M. Li X. Huang Q. Xie X. (2024). Deciphering resveratrol's role in modulating pathological pain: from molecular mechanisms to clinical relevance. Phytother. Res. 38, 59–73. 10.1002/ptr.8021 37795923

[B70] Wang P. J. Yao Q. Zhu D. Yang X. S. Chen Q. J. Lu Q. R. (2023). Resveratrol protects against deoxynivalenol-induced ferroptosis in HepG2 cells. Toxicology 494, 153589. 10.1016/j.tox.2023.153589 37419272

[B71] Wang R. T. Yuan W. D. Li L. Lu F. Zhang L. L. Gong H. F. (2022). Resveratrol ameliorates muscle atrophy in chronic kidney disease via the axis of SIRT1/FoxO1. Phytother. Res. 36, 3265–3275. 10.1002/ptr.7499 35606908

[B72] Wang X. J. Shen T. L. Lian J. Deng K. Qu C. Li E. M. (2023). Resveratrol reduces ROS-induced ferroptosis by activating SIRT3 and compensating the GSH/GPX4 pathway. Mol. Med. 29, 137. 10.1186/s10020-023-00730-6 37858064 PMC10588250

[B73] Wang X. L. Simayi A. Fu J. Zhao X. Xu G. P. (2022). Resveratrol mediates the miR-149/HMGB1 axis and regulates the ferroptosis pathway to protect myocardium in endotoxemia mice. Am. J. Physiol. Endocrinol. Metab. 323, E21–E32. 10.1152/ajpendo.00227.2021 35532075

[B74] Wang Y. M. Wu S. Li Q. Sun H. Y. Wang H. Q. (2023). Pharmacological inhibition of ferroptosis as a therapeutic target for neurodegenerative diseases and strokes. Adv. Sci. (Weinh). 10, e2300325. 10.1002/advs.202300325 37341302 PMC10460905

[B75] Wang H H. Jiang C. Yang Y. K. Li J. H. Wang Y. H. Wang C. N. (2022). Resveratrol ameliorates iron overload induced liver fibrosis in mice by regulating iron homeostasis. PeerJ 10, e13592. 10.7717/peerj.13592 35698613 PMC9188311

[B76] Xia N. Daiber A. Förstermann U. Li H. G. (2017). Antioxidant effects of resveratrol in the cardiovascular system. Br. J. Pharmacol. 174, 1633–1646. 10.1111/bph.13492 27058985 PMC5446570

[B77] Xu K. Chang X. Bai X. Liu H. B. Chen X. B. Chen H. P. (2023). Activation of Nrf2 inhibits ferroptosis and protects against oxaliplatin-induced ototoxicity. Biomed. Pharmacother. 165, 115248. 10.1016/j.biopha.2023.115248 37523980

[B78] Xu L. Y. Botchway B. O. A. Zhang S. G. Zhou J. Y. Liu X. H. (2018). Inhibition of NF-κB signaling pathway by resveratrol improves spinal cord injury. Front. Neurosci. 12, 690. 10.3389/fnins.2018.00690 30337851 PMC6180204

[B79] Xu Y. F. Li K. X. Zhao Y. Zhou L. Liu Y. Zhao J. (2023). Role of ferroptosis in stroke. Cell. Mol. Neurobiol. 43, 205–222. 10.1007/s10571-022-01196-6 35102454 PMC11415219

[B80] Xu Z. Sun X. A. Ding B. Zi M. Ma Y. (2023). Resveratrol attenuated high intensity exercise training-induced inflammation and ferroptosis via Nrf2/FTH1/GPX4 pathway in intestine of mice. Turk. J. Med. Sci. 53, 446–454. 10.55730/1300-0144.5604 37476875 PMC10387861

[B81] Yu W. Chen C. J. Xu C. X. Xie D. Wang Q. Liu W. D. (2022). Activation of p62-NRF2 axis protects against doxorubicin-induced ferroptosis in cardiomyocytes: a novel role and molecular mechanism of resveratrol. Am. J. Chin. Med. 50, 2103–2123. 10.1142/S0192415X22500902 36309811

[B82] Yuan B. Zhao X. D. Shen J. D. Chen S. J. Huang H. Y. Zhou X. M. (2022). Activation of SIRT1 alleviates ferroptosis in the early brain injury after subarachnoid hemorrhage. Oxid. Med. Cell. Longev. 2022, 9069825. 10.1155/2022/9069825 35855863 PMC9288286

[B83] Yuan Y. Mei Z. T. Qu Z. Z. Li G. H. Yu S. T. Liu Y. Q. (2023). Exosomes secreted from cardiomyocytes suppress the sensitivity of tumor ferroptosis in ischemic heart failure. Signal. Transduct. Target. Ther. 8, 121. 10.1038/s41392-023-01336-4 36967385 PMC10040407

[B84] Zeng Y. C. Cao G. D. Lin L. Zhang Y. X. Luo X. Q. Ma X. Y. (2023). Resveratrol attenuates Sepsis-Induced cardiomyopathy in rats through anti-ferroptosis via the Sirt1/Nrf2 pathway. J. Invest. Surg. 36, 2157521. 10.1080/08941939.2022.2157521 36576230

[B85] Zhang T. Chi Y. Q. Kang Y. L. Lu H. Niu H. L. Liu W. (2019). Resveratrol ameliorates podocyte damage in diabetic mice via SIRT1/PGC-1α mediated attenuation of mitochondrial oxidative stress. J. Cell. Physiol. 234, 5033–5043. 10.1002/jcp.27306 30187480

[B86] Zhang W. Qian S. H. Tang B. Kang P. F. Zhang H. Shi C. (2023). Resveratrol inhibits ferroptosis and decelerates heart failure progression via Sirt1/p53 pathway activation. J. Cell. Mol. Med. 27, 3075–3089. 10.1111/jcmm.17874 37487007 PMC10568670

[B87] Zhang Y. P. Xie J. (2024). Induction of ferroptosis by natural phenols: a promising strategy for cancer therapy. Phytother. Res. 38, 2041–2076. 10.1002/ptr.8149 38391022

[B88] Zhang Z. T. Ji Y. Hu N. Yu Q. Q. Zhang X. R. Li J. (2022). Ferroptosis-induced anticancer effect of resveratrol with a biomimetic nano-delivery system in colorectal cancer treatment. Asian. J. Pharm. Sci. 17, 751–766. 10.1016/j.ajps.2022.07.006 36382309 PMC9640689

[B89] Zhao Q. Y. Chang H. R. Chang Y. Z. (2023). Morphological features and the treatment of related diseases of ferroptosis. Prog. Biochem. Biophys. 50, 1286–1295. 10.16476/j.pibb.2022.0287

[B90] Zheng J. S. Conrad M. (2020). The metabolic underpinnings of ferroptosis. Cell. Metab. 32, 920–937. 10.1016/j.cmet.2020.10.011 33217331

[B91] Zheng M. Chen R. F. Zhong H. Y. Lin Q. Y. Wang X. Q. Zhao Z. W. (2012). Side-effects of resveratrol in HepG2 cells: reduced pten and increased bcl-xl mRNA expression. Mol. Med. Rep. 6, 1367–1370. 10.3892/mmr.2012.1077 22971900

[B92] Zhu H. M. Huang J. G. Chen Y. Li X. M. Wen J. Tian M. F. (2022). Resveratrol pretreatment protects neurons from oxygen-glucose deprivation/reoxygenation and ischemic injury through inhibiting ferroptosis. Biosci. Biotechnol. Biochem. 86, 704–716. 10.1093/bbb/zbac048 35357412

[B93] Zhu X. Deng Z. L. Cao Y. J. Zhou Z. H. Sun W. Liu C. (2023). Resveratrol prevents Drp1-mediated mitochondrial fission in the diabetic kidney through the PDE4D/PKA pathway. Phytother. Res. 37, 5916–5931. 10.1002/ptr.8004 37767771

[B94] Zordoky B. N. M. Robertson I. M. Dyck J. R. B. (2015). Preclinical and clinical evidence for the role of resveratrol in the treatment of cardiovascular diseases. Biochim. Biophys. Acta. 1852, 1155–1177. 10.1016/j.bbadis.2014.10.016 25451966

[B95] Zou H. X. Hu T. Zhao J. Y. Qiu B. Q. Zou C. C. Xu Q. R. (2023). Exploring dysregulated ferroptosis-related genes in septic myocardial injury based on human heart transcriptomes: evidence and new insights. J. Inflamm. Res. 16, 995–1015. 10.2147/JIR.S400107 36923465 PMC10010745

[B96] Zou W. Wang X. Xia X. M. Zhang T. T. Nie M. F. Xiong J. (2024). Resveratrol protected against the development of endometriosis by promoting ferroptosis through miR-21-3p/p53/SLC7A11 signaling pathway. Biochem. Biophys. Res. Commun. 692, 149338. 10.1016/j.bbrc.2023.149338 38043156

