# Supplementary figures and images for: Therapeutic potential of resveratrol through ferroptosis modulation: insights and future directions in disease therapeutics
Source: Front Pharmacol. 2024 Sep 25;15:1473939. doi: 10.3389/fphar.2024.1473939 (PMC11461341; doi:10.3389/fphar.2024.1473939)

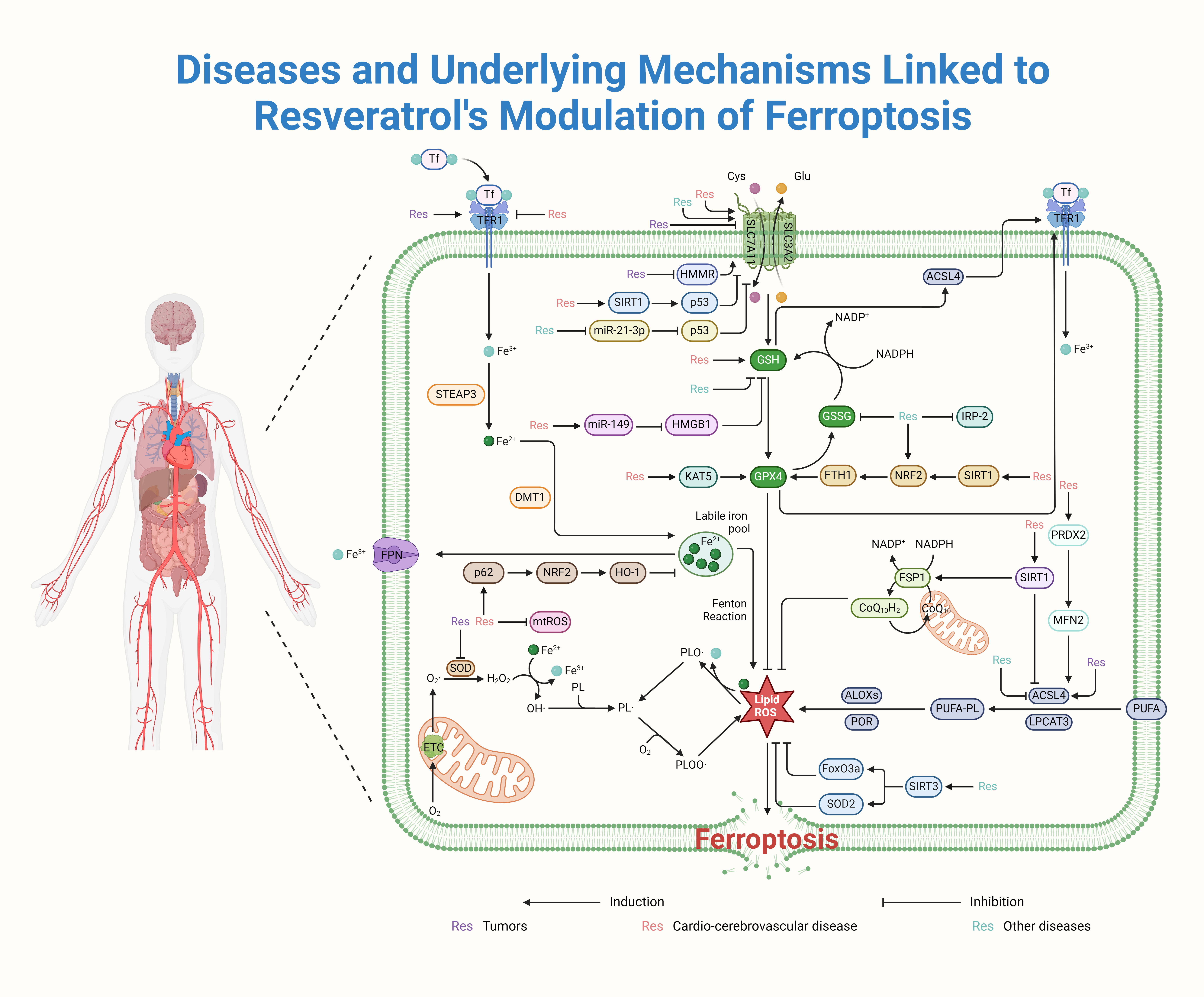

Supplement: Supplementary file 1 [file Image1.TIF]
